# Rosehip Extract-Functionalized Magnesium Hydroxide Nanoparticles and Its Effect on Osteoblastic and Osteoclastic Cells

**DOI:** 10.3390/ma14154172

**Published:** 2021-07-27

**Authors:** Laura Costa Pinho, Thais Francini Garbieri, Liliana Grenho, Marta M. Alves, Pedro Sousa Gomes, Carlos Ferreira Santos, Maria Helena Fernandes, Catarina Santos, Bruno Colaço

**Affiliations:** 1 Department of Animal Science, University of Trás-os-Montes and Alto Douro, 5000-801 Vila Real, Portugal; laurapinho11@gmail.com (L.C.P.); bcolaco@utad.pt (B.C.); 2 Laboratory for Bone Metabolism and Regeneration, Faculty of Dental Medicine, University of Porto, 4200-393 Porto, Portugal; tfgarbieri@usp.br (T.F.G.); lgrenho@fmd.up.pt (L.G.); pgomes@fmd.up.pt (P.S.G.); 3 Department of Biological Sciences, Bauru School of Dentistry, University of São Paulo, Bauru 17012-901, Brazil; cfsantos@fob.usp.br; 4 LAQV/REQUIMTE, University of Porto, 4160-007 Porto, Portugal; 5 Centro de Química Estrutural, Instituto Superior Técnico, Universidade de Lisboa, 1049-001 Lisboa, Portugal; martamalves@tecnico.ulisboa.pt (M.M.A.); catarina.santos@estsetubal.ips.pt (C.S.); 6 EST Setúbal, CDP2T, Instituto Politécnico de Setúbal, Campus IPS, 2910-761 Setúbal, Portugal; 7 CECAV—Animal and Veterinary Research Centre UTAD, University of Trás-os-Montes and Alto Douro, 5000-801 Vila Real, Portugal

**Keywords:** Mg(OH)_2_ nanoparticles, green-synthesis, rosehip extract, osteoblastic differentiation, osteoclastic differentiation, bone metabolism

## Abstract

Considering the role of magnesium in bone metabolism and the increasing relevance of plant-mediated green-synthesis, this work compares the bone cytocompatibility of magnesium hydroxide nanoparticles (NPs) produced by using pure water, Mg(OH)_2_, or a rosehip (RH) aqueous extract, Mg(OH)_2_RH. The NPs were evaluated for dose- and time-dependent effects on human osteoblastic and osteoclastic response, due to the direct involvement of the two cell types in bone metabolism. Mg(OH)_2_ NPs presented nanoplatelet-like morphology (mean diameter ~90 nm) and a crystalline structure (XRD analysis); the RH-mediated synthesis yielded smaller rounded particles (mean diameter <10 nm) with decreased crystallinity. On the ATR–FTIR spectra, both NPs presented the characteristic Mg-OH peaks; Mg(OH)_2_RH exhibited additional vibration bands associated with the presence of phytochemicals. On osteoblastic cells, NPs did not affect cell growth and morphology but significantly increased alkaline phosphatase (ALP) activity; on osteoclastic cells, particles had little effect in protein content, tartrate-resistant acid phosphatase (TRAP) activity, percentage of multinucleated cells, and cell area. However, compared with Mg(OH)_2_, Mg(OH)_2_RH increased osteoblastic differentiation by inducing ALP activity and promoting the expression of Runx2, SP7, Col1a1, and ALP, and had a negative effect on the expression of the osteoclastic genes NFATC1, CA2, and CTSK. These observations suggest the potential usefulness of Mg(OH)_2_RH NPs in bone regeneration.

## 1. Introduction

Bone is a mineralized connective tissue with well-recognized mechanical and metabolic functions. Besides giving support and protection to the body, it can also store important ions such as calcium, phosphate, magnesium, and others, and harbor in its internal cavities bone marrow production and hematopoietic cell development [1,2]. Bone tissue is majorly composed of a calcified bone matrix and four types of bone cells. Osteoclasts are of hematopoietic origin while osteoblasts, osteocytes, and bone lining cells are all originated from the differentiation of mesenchymal stem cells (MSCs) [2]. Being a dynamic tissue, it is constantly in a remodeling process through the concerted actions of the bone cells, together with interactions with other cells present in the bone microenvironment, e.g., endothelial and immune cells [3]. Bone remodeling is accomplished by the resorption of bone by the osteoclasts with the formation of a resorption lacuna, and the subsequent filling of this lacuna with newly synthesized bone by the osteoblasts, in order to maintain a mechanical and metabolic healthy tissue [4,5].

Although bone can regenerate itself, in severe cases of bone loss or established defects, appropriate cost-effective and efficient biomaterial-mediated approaches are required to achieve complete healing—regenerating tissue structure, volume, and functions [6]. Bone regenerative biomaterials have to fulfill essential prerequisites such as biocompatibility, osteoinductivity, and adequate physicochemical properties to provide an optimal environment for cell proliferation and differentiation [7,8]. Magnesium-based materials bear a great potential for bone applications, as filling materials or by being added to hydroxyapatite nanocomposites [9], implant scaffolds [10], alloys [11], bioceramics, and glass [12], to induce bone regeneration and improve their mechanical properties [13].

Magnesium (Mg) is naturally biocompatible as it exists abundantly in the human body, is mostly stored in bone (>50%) [14], and has a key role in bone metabolism. In addition, Mg is easily excreted when in excess [15]. This ion plays important roles both extracellularly, being found in the ionized form or bound to proteins or anions, and intracellularly, co-factoring more than 300 enzymatic reactions involved in the synthesis of proteins, nucleic acids and lipids [16], energy production pathways, DNA stabilization, and regulating the transport of calcium, potassium, and sodium ions [15,17,18]. Results from in vitro and in vivo studies suggest a positive effect of Mg^2+^ and Mg-containing materials on bone applications, by favoring osteoblastic behavior [19,20,21,22] and bone formation [23]. Accordingly, metal hydroxides such as Mg(OH)_2_ nanoparticles (NPs) hold great potential for bone regeneration owing to their osteoinductive effects and anti-inflammatory activity [24]. Furthermore, these NPs have been successfully deployed as inorganic antibacterial agents, due to their unique antibacterial properties, high stability, and low cost of production [25,26,27].

Plant-mediated green synthesis of nanoparticles is becoming increasingly relevant as an alternative to conventional chemical synthesis as a reliable, sustainable, and eco-friendly methodology. It has been quite successful in the synthesis of metal and metal oxide NPs [28]. Plant phytochemicals may play a dual role by acting as both reducing and stabilizing agents in NPs synthesis. Additionally, the presence of bioactive molecules endows NPs with appellative functional properties [28,29]. Green-synthesized MgO NPs have been reported in several studies and applications [29], but this is not the case of Mg(OH)_2_ NPs. In addition, the bone application potential of these NPs has received little attention, compared with that of MgO NPs.

Rosehip has long been used for medicinal purposes due to the high content of bioactive compounds [30]. It has a recognized antioxidant activity, presenting high levels of polyphenols, Vitamins C, E, B, and carotenoids, which demonstrate synergistic effects [30,31]. The antioxidant activity has been related to the reported positive effects in conditions of bone loss associated with increased oxidative stress leading to excessive bone resorption, i.e., osteoarthritis, rheumatoid arthritis, and osteoporosis [32,33].

In this context, this work compares the bone cytocompatibility of Mg(OH)_2_ NPs produced by a conventional chemical process or a green synthesis mediated process, using a rosehip aqueous extract. The two NPs were evaluated for the dose- and time-dependent effects on human osteoblastic and osteoclastic response, as the two cell types are directly involved in bone metabolism during bone remodeling and regeneration. The aim is to achieve more comprehensive and integrative information on the cytocompatibility of these nanoparticles on bone cells and, additionally, the potential added value of the green synthesis process.

## 2. Materials and Methods

### 2.1. Synthesis and Physicochemical Characterization of Mg(OH)_2_ Nanoparticles

Commercial dried rosehip cinhorrods (RH) were used to produce magnesium hydroxide nanoparticles. To guarantee a complete extraction of the bioactive compounds and the inactivation of enzymatic activities, 5 g of RH was mixed with 250 mL of de-ionized water and boiled. The RH extract obtained was cooled down to room temperature and filtered. Mg(OH)_2_ NPs were synthesized using 1 g of magnesium nitrate (Mg(NO_3_)_2_·6H_2_O, Sigma-Aldrich, St. Louis, MO, USA) as the precursor, and the process was carried out at room temperature in pure water or recurring to a green synthesis using a 75% aqueous rosehip (RH) extract. Two NPs, respectively Mg(OH)_2_ and Mg(OH)_2_RH, were obtained after addition of a KOH solution (5M). Finally, the NPs were collected and washed with deionized water by centrifuging twice at 800 rpm for 10 min. These NPs were then dried at room temperature in a desiccator.

The size and shape of NPs were characterized by scanning electron microscopy (SEM) using a JEOL-JSM7001F apparatus (JEOL, Tokyo, Japan). To increase the conductivity of Mg(OH)_2_ NPs, a thin coating of conductive chromium or gold/palladium (Polaron E-5100) was applied. The crystallinity of NPs was identified by X-ray diffraction (XRD) using a D8 Advance Bruker AXS (Bruker, Billerica, MA, USA). The chemical characterization of Mg(OH)_2_ and Mg(OH)_2_RH NPs was also performed with Fourier transformed infrared spectroscopy (FTIR) using a Nicolet (Thermo Electron, Waltham, MA, USA) spectrometer with an attenuated total reflectance (ATR) apparatus.

### 2.2. Cell Cultures

#### 2.2.1. MG-63 Cell Cultures and Exposure to Mg(OH)_2_ Nanoparticles

MG-63 (ATCC^®^CRL-1427™; ATCC, Manassas, VA, USA) cells were cultured (2 × 10^4^ cells/cm^2^) in RPMI-1640 medium supplemented with 10% fetal bovine serum (FBS), 100 IU/mL penicillin, 100 µg/mL streptomycin, and 2.5 µg/mL amphotericin B (basal medium; all reagents from Gibco, Waltham, MA, USA) in 96-well plates (Falcon, New York, NY, USA) at 37 °C, 95% humidity, and 5% CO_2_ atmosphere. After 24 h, the medium was exchanged for fresh basal medium (negative control) or basal medium supplemented with 10 nM dexamethasone and 50 µg/mL ascorbic acid (osteogenic medium—positive control; all reagents from Sigma-Aldrich, St. Louis, MO, USA), or exposed to Mg(OH)_2_ NPs prepared in basal medium (1, 10, and 100 µg/mL). This concentration range was selected from a preliminary experiment showing that levels higher than 500 μg/mL caused dose-dependent deleterious effects in cell viability and, further, osteoblastic inductive effects on ALP activity were observed at levels around 10 μg/mL. Control and NP-exposed cultures were grown for 1, 3, and 6 days, and characterized for metabolic activity (MTT assay), alkaline phosphatase (ALP) activity, gene expression of osteoblastic markers, observation by light microscopy after ALP staining, and fluorescence microscopy after immunostaining for F-actin cytoskeleton and nucleus.

#### 2.2.2. THP-1 Cell Cultures and Exposure to Mg(OH)_2_ Nanoparticles

THP-1 (ATCC^®^TIB-202™; ATCC, Manassas, VA, USA) monocytic cells were suspended (1.25 × 10^5^ cells/cm^2^) in RPMI-1640 medium supplemented with 10% fetal bovine serum (FBS), 100 IU/mL penicillin, 100 µg/mL streptomycin, 2.5 µg/mL amphotericin B, and 0.05 mM 2-mercaptoethanol (basal medium; all reagents from Gibco, Waltham, MA, USA) in 24-well plates (Falcon, New York, NY, USA). For differentiation into macrophage-like cells, 100 ng/mL phorbol 12-myristate 13-acetate (PMA, Sigma-Aldrich, St. Louis, MO, USA) was added to the medium, and the plates were incubated at 37 °C, 95% humidity, and 5% CO_2_ atmosphere for 48 h. Then, the cell medium was changed for basal medium (negative control) and the osteoclastic differentiation cultures were supplemented with 50 ng/mL of macrophage colony-stimulating factor (M-CSF) and 50 ng/mL of receptor activator of nuclear factor kappa-B ligand (RANKL) (both from PeproTech, London, UK)—positive control, or exposed to Mg(OH)_2_ NPs (1, 10, and 100 µg/mL), and cultured for 1 and 6 days. For comparative purposes, the tested concentration range was similar to that used to analyze the osteoblastic response. Cell response was evaluated for total protein content, tartrate-resistant acid phosphatase (TRAP) activity, gene expression of osteoclastic markers, and observation by light microscopy after TRAP staining, and fluorescence microscopy after immunostaining of F-actin cytoskeleton and nucleus.

### 2.3. Characterization of Cell Response

#### 2.3.1. Metabolic Activity

Metabolic activity was assessed through the MTT assay on days 1, 3, and 6 for MG-63 cell cultures and at days 1 and 6 for THP-1 cell cultures. MTT (5 mg/mL, Sigma-Aldrich, St. Louis, MO, USA) was added and the culture was incubated for 3 h at 37 °C. Reduction of MTT (3-(4,5-Dimethylthiazol-2-yl)-2,5-Diphenyltetrazolium Bromide) led to the formation of violet-blue formazan crystals by metabolic active cells. Then, the culture medium was removed and dimethyl sulfoxide (DMSO, Panreac, Darmstadt, Germany) was added (room temperature, 15 min) to dissolve the formazan salts. Absorbance was measured at λ = 550 nm in a microplate reader (Synergy HT, Biotek, Winooski, VT, USA).

#### 2.3.2. Total Protein Content

Total protein content was quantified in MG-63 cell cultures (days 1, 3, and 6) and THP-1-derived cell cultures (days 1 and 6). Cell lysates (Triton X-100 0.1%, 30 min) were evaluated using the *DC*^TM^ Protein Assay (BioRad, Hercules, CA, USA), according to the manufacturer’s instructions.

#### 2.3.3. Alkaline Phosphatase Activity and Staining

ALP activity of MG-63 cell cultures was evaluated on days 1, 3, and 6 in cell lysates (Triton X-100 0.1%, 30 min) by the hydrolysis of p-nitrophenyl phosphate (p-NPP, 25 mM, Sigma-Aldrich, St. Louis, MO, USA) in an alkaline buffer (pH 10.3, 37 °C, 1 h). The reaction was stopped with NaOH 5 M and the product (p-nitrophenol) [34] was measured at λ = 400 nm in a microplate reader (Synergy HT, Biotek, Winooski, VT, USA). Results were normalized to total protein content and expressed as nanomoles of p-nitrophenol per microgram of protein (nmol/μg protein).

For ALP staining, cultures were fixed in glutaraldehyde 1.5% (TAAB) in sodium cacodylate buffer 0.14 M (Sigma-Aldrich) for 15 min. Fixed cultures were incubated in a filtered solution of sodium naphthyl phosphate (2 mg/mL, Sigma-Aldrich) and Fast Blue RR (2 mg/mL, Sigma-Aldrich) in Tris buffer solution 0.1 M, pH 10 for 1 h and protected from light. Stained cultures were observed by light microscopy (Primo Vert™ Inverted Microscope, Carl Zeiss, Jena, Germany). The ALP presented a brown to black staining.

#### 2.3.4. Tartrate-Resistant Acid Phosphatase Activity and Staining

TRAP activity was evaluated (days 1 and 6) in cell lysates of THP-1-derived cell cultures (Triton X-100 0.1%, 30 min) by the hydrolysis of p-nitrophenyl phosphate 25 mM (p-NPP) in tartaric acid buffer (0.04 M tartaric acid and 0.09 M citrate, pH 4.8) at 37 °C for 1 h. The reaction was stopped with NaOH 5 M and absorbance was measured at λ = 400 nm in a microplate reader (Synergy HT, Biotek, Winooski, VT, USA). Results were normalized to total protein content and expressed as nanomoles of p-nitrophenol per microgram of protein (nmol/μg protein).

TRAP staining (days 1 and 6) was performed in fixed cultures (as described above) using the Leukocyte Acid Phosphatase (TRAP) kit (Sigma-Aldrich, St. Louis, MO, USA) according to the manufacturer’s instructions. Stained cultures were evaluated in Primo Vert™ Inverted Microscope (Carl Zeiss, Jena, Germany) for the presence of TRAP(+) cells, stained purple.

#### 2.3.5. Immunostaining of F-Actin Cytoskeleton and Nucleus

MG-63 cell cultures and THP-1-derived cell cultures exposed to Mg(OH)_2_ NPs (10 µg/mL) were fixed (formaldehyde 3.7%, 10 min), permeabilized (Triton X-100 0.1% in PBS, 15 min, room temperature), and incubated with bovine serum albumin (BSA 1% in PBS, 30 min, Sigma-Aldrich, St. Louis, MO, USA) to reduce non-specific coloring. Cultures were stained for F-actin cytoskeleton with Alexa Fluor^®^ 488 phalloidin (1:100, 30 min, Molecular Probes, Eugene, OR, USA), and nucleus with Hoechst (8 µg/mL, 10 min, Enzo, New York, NY, USA). Images were obtained using the Celena S digital imaging system (Logos Biosystems, Anyang, South Korea). Cell area and the number of multinucleated cells (≥3 nuclei) were evaluated using the measure tool of ImageJ software.

#### 2.3.6. Real-Time Quantitative Polymerase Chain Reaction (RT-qPCR)

Cell cultures exposed to Mg(OH)_2_ NPs (10 µg/mL) were characterized by real-time quantitative polymerase chain reaction (RT-qPCR) to assess the osteogenic differentiation of MG-63 cells and the osteoclastogenic differentiation of THP-1-derived cells on day 1.

Total RNA was extracted using the TRIzol™ reagent (Invitrogen, Waltham, MA, USA) and reverse-transcribed into complementary DNA (cDNA) with the NZY First-Strand cDNA Synthesis Kit (Nzytech, Lisbon, Portugal), all according to the manufacturer’s instructions. The expression of the target genes was quantitatively determined on RT-PCR equipment (CFX96, BioRad) using iQTM SYBR^®^ Green Supermix (BioRad, Hercules, CA, USA).

All genes were normalized to the reference gene (GADPH, BioRad) and are described in Table 1.

### 2.4. Statistical Analysis

All data were obtained from three separate experiments, each one performed in triplicate, and expressed as mean values ± standard deviation. Statistical analysis was performed using the IBM^®^ SPSS^®^ Statistics 25. Data normality was assessed by the Shapiro–Wilk test. Regarding normal datasets, one-way analysis of variance (ANOVA) was performed, followed by the post hoc Tukey test. For non-parametric datasets, the Kruskal–Wallis test was performed, followed by multiple comparisons using Dunn’s tests. For both, *p*-values ≤ 0.05 were considered significant.

## 3. Results

### 3.1. Mg(OH)_2_ NPs

**Figure 1:**
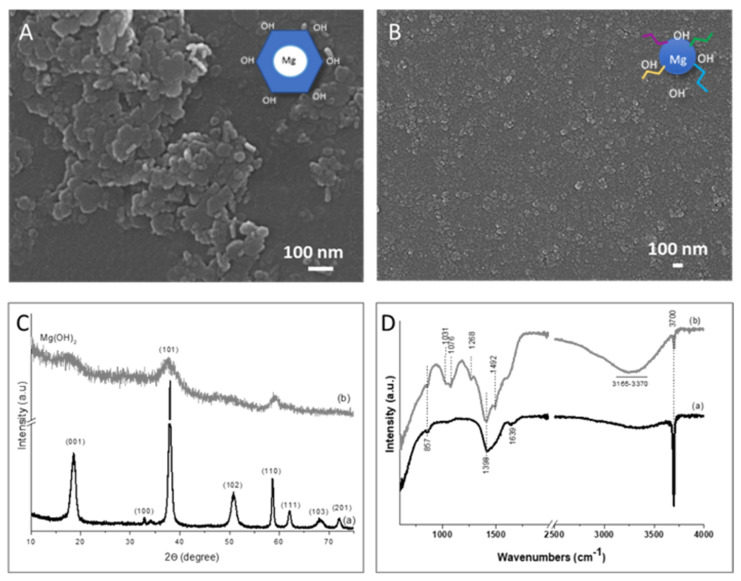
Mg(OH)_2_ NPs synthesized using water or a RH extract. (**A**) and (**B**)—SEM image of Mg(OH)_2_ NPs (**A**) and Mg(OH)_2_RH NPs (**B**); (**C**)—XRD diffractograms of Mg(OH)_2_ NPs (a) and Mg(OH)_2_RH NPs (b); and (**D**)—ATR–FTIR spectrum of Mg(OH)_2_ NPs (a) and Mg(OH)_2_RH NPs (b).

The morphology of synthesized Mg(OH)_2_ and Mg(OH)_2_RH NPs was observed by SEM and displayed in Figure 1. A nanoplatelet-like shape morphology with a mean diameter of 90 nm was observed for Mg(OH)_2_ NPs (Figure 1A), while a spherical-like shape with a size less than 10 nm was observed for Mg(OH)_2_RH (Figure 1B). To evaluate the crystalline phases present on the NPs, an XRD analysis was performed (Figure 1C). In the Mg(OH)_2_ NPs (Figure 1C (a)), diffraction sharp peaks were clearly observed, especially the intense (001) diffraction plane, characteristic of platelet-like particles. On the other hand, in the Mg(OH)_2_RH (Figure 1C (b)), an overall reduction in crystallinity was observed through a broadening and decreased intensity of the sharped Mg(OH)_2_ peaks. The ATR–FTIR analysis was performed in order to evaluate the presence of phytochemicals in Mg(OH)_2_RH (Figure 1D). In both NPs, the presence of Mg(OH)_2_ characteristic peaks is clear, namely, the Mg-OH peaks (3700 cm^−1^, 1639, and 1398 cm^−1^). The presence of phytochemicals derived from the RH extract on Mg(OH)_2_RH NPs was confirmed by the additional vibration bands observed at 1031, 1076, 1268, and 1492 cm^−1^ in ATR–FTIR spectra (Figure 1D (b)).

### 3.2. Effect of Mg(OH)_2_ NPs in the Osteoblastic Behavior

MG-63 cells cultured in basal conditions were exposed to Mg(OH)_2_ NPs, 1 to 100 μg/mL, for periods up to 6 days, and cell behavior was analyzed for viability/proliferation, immunostaining of F-actin cytoskeleton, ALP activity and staining, and expression of osteoblastic genes. Results were compared with those in cultures performed in basal conditions (basal, control) and upon osteogenic induction (osteogenic, positive control).

#### 3.2.1. Metabolic Activity/Proliferation and Morphology

**Figure 2:**
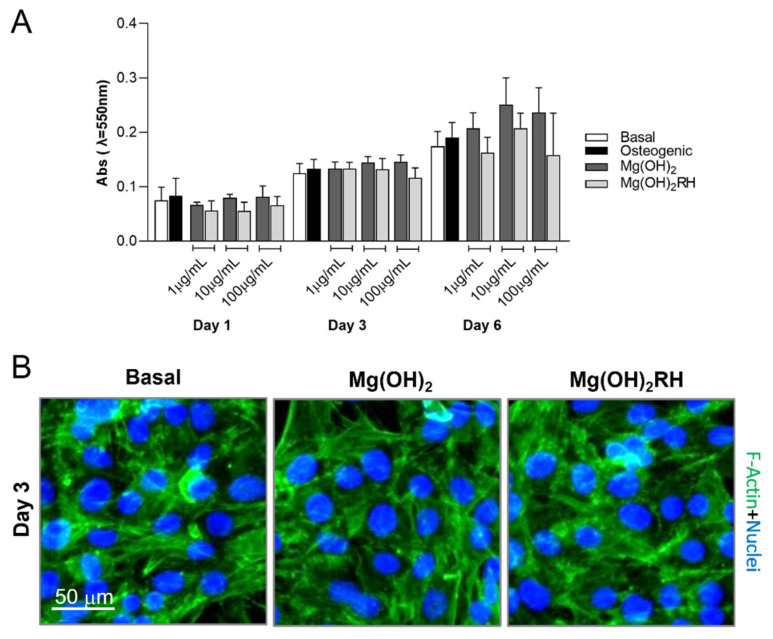
(**A**)—cell viability/proliferation (MTT assay) of MG-63 cells cultured in basal and osteogenic conditions, and exposed to Mg(OH)_2_ and Mg(OH)_2_RH NPs at 1, 10, and 100 µg/mL, for 1, 3, and 6 days. (**B**)—immunostaining of F-actin cytoskeleton (green) and nucleus (blue) of cultures grown in basal conditions and treated with the NPs (10 µg/mL) for 3 days.

In all cultures, metabolic activity increased throughout the culture time, Figure 2A, which translates into an increase in cell proliferation. Cells exposed to the NPs presented values similar to control cultures within the tested concentration range, for each time point. However, on day 6, cultures treated with 10 μg/mL NPs showed a tendency for increased values. The two NPs presented similar behavior.

Cultures were also stained for F-actin cytoskeleton and nucleus, and illustrative images are shown in Figure 2B. Identical appearance was observed in all conditions (controls and NPs-treated cultures), namely, an elongated morphology with normal cytoskeleton organization, abundant cell-to-cell contact, and prominent nucleus.

#### 3.2.2. ALP Activity and Expression of Osteoblastic Genes

**Figure 3:**
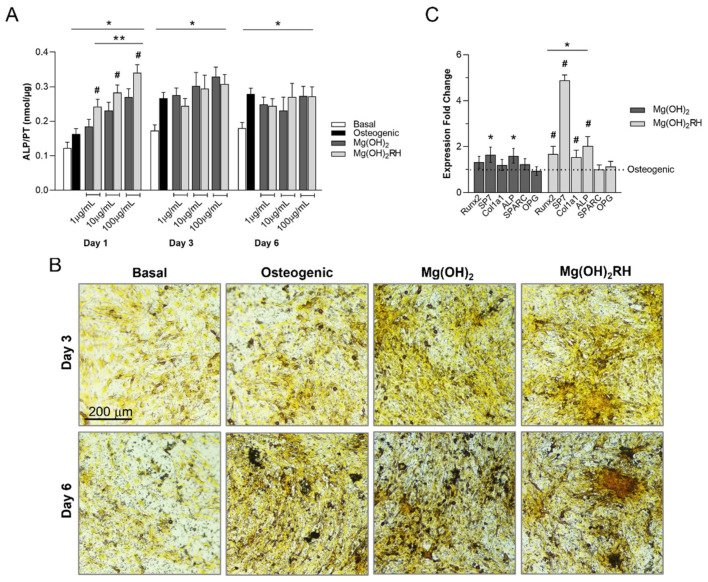
(**A**)—ALP activity of MG-63 cells cultured in basal and osteogenic conditions and exposed to Mg(OH)_2_ and Mg(OH)_2_RH NPs (1, 10, and 100 µg/mL) for 1, 3, and 6 days. * Significantly different from cultures grown in basal medium; ** significantly different from cultures grown in the osteogenic medium; and # significantly different from Mg(OH)_2_ NPs. (**B**)—histochemical staining of ALP in control and NPs-exposed (10 μg/mL) cultures for 3 and 6 days. (**C**)—expression of osteoblastic genes in osteogenic and NPs-treated cultures, at day 1. * Significantly different from the cultures performed in osteogenic conditions; and ^#^ significantly different from the cultures exposed to Mg(OH)_2_.

In all cultures, ALP activity increased from day 1 to day 3, stabilizing afterward. In osteogenic conditions, values were significantly higher than those observed in basal medium. Exposure to the NPs induced ALP activity. On day 1, overall, NPs-treated cultures presented significantly increased values, compared with those grown in basal and also in osteogenic conditions. Furthermore, Mg(OH)_2_RH NPs elicited higher ALP induction than Mg(OH)_2_ NPs. On days 3 and 6, ALP values of NPs-treated cultures were similar to those found in the osteogenic medium and thus significantly higher than that observed in basal conditions. Differences between the two particles were not verified. Results are presented in Figure 3A.

Figure 3B shows representative images for the histochemical staining of ALP in control cultures and those exposed to the NPs at 10 μg/mL. No apparent deleterious effects were observed in the presence of NPs. Compared with the controls, exposed cultures presented a similar pattern of cell growth and organization. Cells proliferated in cellular agglomerates that exhibited stronger staining for the presence of ALP. Images suggest that the cultures exposed to the nanoparticles displayed an appearance closer to the cultures grown in osteogenic conditions.

Mg(OH)_2_ and Mg(OH)_2_RH NPs-treated cultures (10 µg/mL) were compared with the osteogenic condition for the expression of several osteoblastic genes, Figure 3C. Mg(OH)_2_ NPs increased the expression of SP7 and ALP, whereas Mg(OH)_2_RH NPs induced the gene expression of Runx-2, SP7, Colla1, and ALP. Furthermore, expression of these genes was also higher compared with that observed in the cultures exposed to Mg(OH)_2_ NPs. The expression of SPARC and OPG was similar in all conditions.

### 3.3. Effect of Mg(OH)_2_ NPs in Osteoclastic Behavior

Mg(OH)_2_ NPs were evaluated for their effect in the osteoclastic differentiation of THP-1-derived macrophages. Cell response was compared with that observed in the cultures performed in the presence of the osteoclastogenic inducers M-CSF and RANKL (positive control). Results for the cultures performed in basal conditions are also shown.

#### 3.3.1. Total Protein Content, Metabolic Activity, and Cell Morphology

**Figure 4:**
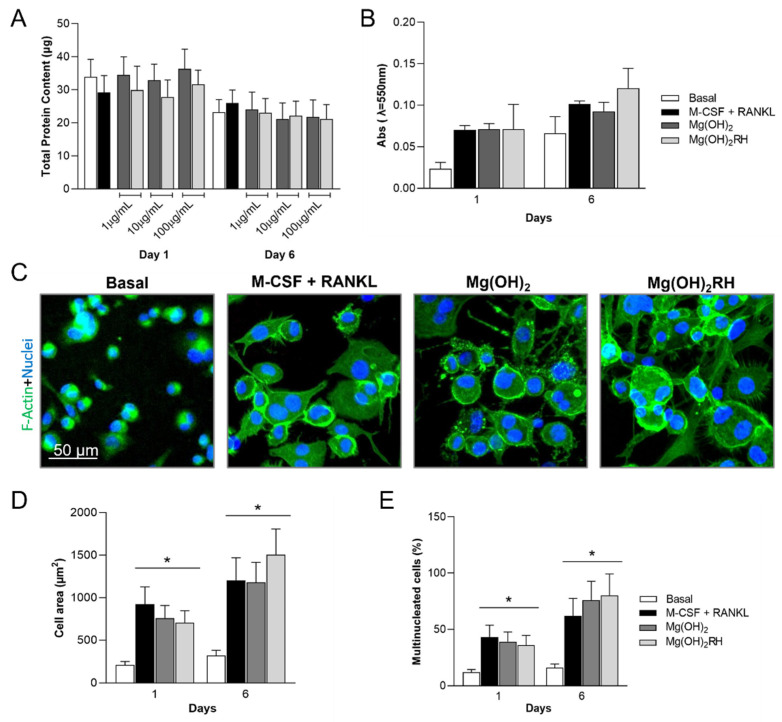
(**A**)—total protein content of THP-1-derived cells cultured in basal medium, in the presence of M-CSF and RANKL, and exposed to Mg(OH)_2_ and Mg(OH)_2_RH NPs (1, 10 and 100 µg/mL) for 1 and 6 days; (**B**)—cell viability (MTT assay) of THP-1 cells cultured in basal medium, in the presence of M-CSF and RANKL, and exposed to Mg(OH)_2_ and Mg(OH)_2_RH NPs at 100 µg/mL, for 1 and 6 days. (**C**)—immunostaining of F-actin cytoskeleton (green) and nucleus (blue) of cultures grown in basal conditions, in the presence of M-CSF and RANKL, and treated with the NPs (10 µg/mL) for 1 day; (**D**)—cell area; and (**E**)—percentage of multinucleated cells. * Significantly different from the cultures grown in basal medium.

Total protein content of THP-1-derived cells exposed to the NPs was similar to that of control cultures (basal and osteoclastogenic-induced), at days 1 and 6, Figure 4A.

Cells metabolic activity (Figure 4B) was assessed in the presence of the highest NPs concentration (100 µg/mL), and was similar to that measured in the cultures treated with M-CSF and RANKL. Cell morphology was evaluated in cultures stained for F-actin cytoskeleton and nucleus, Figure 4C (1-day cultures). In basal conditions, cells were small and displayed a rounded shape. Significant changes were induced in the presence of M-CSF and RANKL. Exposure to the NPs had a similar effect. Cells kept the round appearance but increased in size, showed well-defined actin rings, and a high percentage of multinucleated cells were observed in the cultures. Furthermore, the images were analyzed for the quantification of the cell area (Figure 4D) and the number of multinucleated cells (Figure 4E). Cell area increased from day 1 to day 6 in all situations. A similar pattern was observed for the % of multinucleated cells. In both parameters, no significant differences were observed between the positive control and the NPs-treated cultures, or between the two types of particles.

#### 3.3.2. TRAP Activity and Expression of Osteoclastic Genes

**Figure 5:**
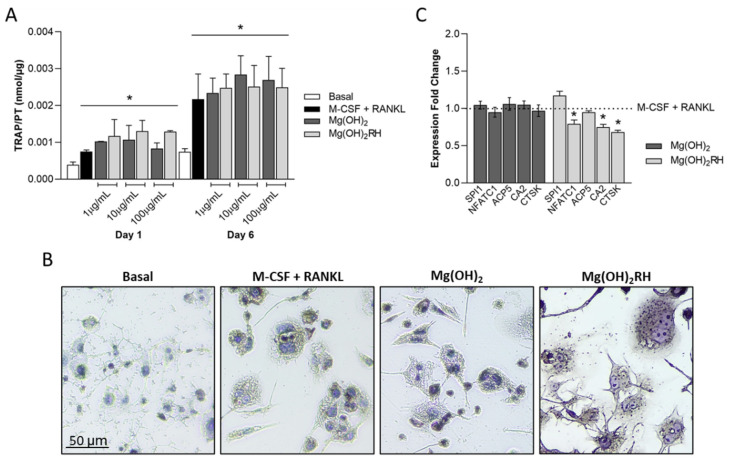
(**A**)—TRAP activity of THP-1-derived cells cultured in basal medium, in the presence of M-CSF and RANKL, and exposed to Mg(OH)_2_ and Mg(OH)_2_RH NPs (1, 10, and 100 µg/mL) for 1 and 6 days. * Significantly different from the cultures grown in basal medium. (**B**)—histochemical staining of TRAP in control cultures and exposed to the NPs (10 µg/mL) for 6 days; (**C**)—expression of osteoclastic genes in cultures treated with the NPs (10 µg/mL) for 1 day. * Significantly different from the cultures grown in the presence of M-CSF and RANKL.

TRAP activity was analyzed on days 1 and 6. In basal conditions, values were very low, increasing significantly in the treated cultures. The differentiation factors (M-CSF and RANKL) and the NPs induced similar behavior. In both cases, TRAP activity increased from day 1 to day 6, Figure 5A. Cultures were also stained for the presence of TRAP, Figure 5B (6-day cultures). All treated cultures exhibited positive staining. Further, cell morphology is in line with that on the fluorescence images of F-actin stained cultures, i.e., displaying a rounded shape and showing several nuclei.

Mg(OH)_2_ and Mg(OH)_2_RH NPs-treated cultures were compared with cells cultured in the presence of M-CSF and RANKL (positive control) for the expression of several osteoclastic genes, Figure 5C. Mg(OH)_2_ did not cause any significant effect in gene expression. Comparatively, the expression of NFATC1, CA2, and CTSK was significantly lower in the cells exposed to Mg(OH)_2_RH NPs. In all conditions, cultures displayed similar expression of SPI1 and ACP5.

## 4. Discussion

Metal-based nanomaterials are commonly used for various biomedical applications including bone tissue regeneration. These materials provide a wide range of advantages due to their physiological functions and physicochemical properties that favor biological interactions [8,13]. Magnesium ion (Mg^2+^) participates in osteoblastic proliferation, differentiation, activity, and bone formation, being an important natural mineral for bone metabolic activities [13]. Nanoparticulate Mg-formulations, due to the high surface/volume ratio, further potentiate the advantages of these materials [35].

This study reports the response of human osteoblastic and osteoclastic cells to Mg(OH)_2_ nanoparticles, aiming to integrate information on the cells responsible for bone formation and resorption, respectively. Further, Mg(OH)_2_ NPs were produced by two processes, i.e., involving synthesis in pure water or a green process using an aqueous extract of the flavonoid-rich rosehip (RH).

The two synthesized Mg(OH)_2_ particles presented nanometer size and the characteristic Mg-OH peaks (3700 cm^−1^, 1639, and 1398 cm^−1^) on the ATR–FTIR spectra [36]. Furthermore, the presence of phytochemicals derived from the RH extract on Mg(OH)_2_RH NPs was confirmed by the additional vibration bands observed at 1031, 1076, 1268, and 1492 cm^−1^. The two particles differed in morphology and size (SEM observation) and crystallinity (XRD analysis). Compared with the platelet-shaped and crystalline Mg(OH)_2_ NPs, the RH-mediated synthesis yielded smaller rounded particles with decreased crystallinity. Thus, the presence of derived phytochemicals from the RH extract modified the morphology and decreased the size of Mg(OH)_2_ NPs. It is known that the shape of a NP is determined by the relative growth rates of individual faces of the crystal that constitutes the NP, which is strongly dependent on the precipitate solution pH and ion’s nature [37,38]. Possibly a high concentration of organic molecules was adsorbed or interacted on the (001) NPs crystal faces during the nucleation, given to the rising to small spherical NPs instead of large plate-like NPs [26,39]. As expected, the observed decrease in particle size and probably the high absorption of phytochemicals due to the large surface area resulted in a significant decrease in crystallinity.

Bone cell response to Mg(OH)_2_ and Mg(OH)_2_RH NPs was addressed with two human cell lines, as an alternative to primary cells [40], namely, the osteoblastic MG-63 cell line and the osteoclastic-differentiated THP-1 monocytes. These cell lines show some matching behavior with normal cells, allowing the analysis of common features of bone cells, and present phenotypic stability and high cell availability, contributing to high reliability when comparing different studies [41,42,43].

MG-63 cells exhibit a variety of osteoblastic markers and are sensitive to hormonal response, being suited to be used as an in vitro model for testing biomaterials for bone applications [43,44,45]. In the present work, these cells were cultured in basal conditions and in an osteogenic medium, namely, in the presence of ascorbic acid and dexamethasone, two molecules routinely used to induce osteoblastic differentiation. Ascorbic acid is required for the synthesis of collagen type I, the main component of the extracellular matrix, and dexamethasone induces and regulates Runx2 expression, promoting osteogenic differentiation [46]. Results showed that cells grown in osteogenic medium presented significantly higher ALP activity and histochemical staining (Figure 3), attesting to the responsiveness of these cells to the surrounding culture conditions. Thus, cultures performed in basal conditions and osteogenic conditions were used as negative and positive controls.

The NPs in the concentration range of 1 to 100 μg/mL did not affect cell viability/proliferation (Figure 2), which was in line with that previously observed [47]. Regarding differentiation markers, both NPs greatly induced ALP activity (Figure 3). This induction occurred soon after the addition of the particles, being particularly evident after 1-day exposure, and was even higher than that observed in osteogenic conditions (positive control). The stimulatory effect was still observed after 3 and 6 days, and ALP activity was similar to that in the cultures grown in osteogenic conditions. Furthermore, the exposure to the NPs clearly improved the organization and maturation of the cell layer, evidenced by the presence of defined tridimensional cellular agglomerates that stained intensively for ALP, compared with that on basal conditions. This positive effect was most probably associated, at least in part, to the Mg-particle composition, and this observation is in line with a variety of previous studies [19,20,21,22,48]. A recent review summarized the eventual underlying mechanisms, stressing that Mg ions appear to act in the all proliferation/osteoblastic differentiation pathway, by inducing early and late gene expression (Runx2, BMP-2, ALP, OCN, OPN) associated with a variety of osteogenic signaling pathways (integrin α2, integrin β1, FAK, ERK1/2, Notch1) [49], a process that is dose-dependent [50,51].

Although both NPs clearly induced ALP activity, at 1-day exposure, Mg(OH)_2_RH NPs exhibited a higher effect. These inductive effects were further investigated by evaluating the expression of some osteoblastic genes, comparing with that observed in osteogenic-induced cultures (Figure 3). Both particles induced the expression of SP7 (Osterix) and ALP. However, the green-synthesized particles further increased the expression of Runx2 and collagen type I. Comparing the two NPs, Mg(OH)_2_RH induced the genes coding for the early and late transcription factors, respectively, Runx2 and Osterix, collagen type I, the main component of the bone extracellular matrix, and ALP, the enzyme needed for the initiation of the matrix mineralization [52]. Expression of OPG, a regulatory inhibitor of osteoclastogenesis in the osteoblast-osteoclast RANKL/RANK/OPG signaling [53] appeared to not be affected, suggesting that the interaction between the two cell types is probably not significantly affected. Several factors might account for the higher osteoblastic inductive effect of Mg(OH)_2_RH NPs. As previously mentioned, these particles were much smaller (with a greater surface area) and less crystalline compared with Mg(OH)_2_ NPs, two characteristics contributing to a higher solubility and, possibly, to the presence of more appropriate levels of Mg ions. Additionally, the presence of phytochemicals in the particles, as confirmed on the ATR–FTIR spectrum (Figure 1), is also expected to play a role. Rosehip bears high levels of bioactive molecules such as flavonoids, carotenoids, and ascorbic acid [32,54,55,56]. These compounds have been reported to preserve bone health and prevent metabolic-related bone loss conditions [32,57,58,59,60], improving osteoblastogenesis by reducing the effects of oxidative stress or chronic low-grade inflammation [57,59].

Mg(OH)_2_ and Mg(OH)_2_RH NPs were also assessed for their effect on osteoclast behavior, using the THP-1 monocyte cell line, that frequently serves as a human osteoclast model [61,62,63]. THP-1 cells were first treated with PMA to be driven into macrophages and, subsequently, with the osteoclastogenic factors M-CSF and RANKL to achieve osteoclastic differentiation (positive control), following a previously described methodology [62]. These conditions allow the development of cells with osteoclastic features. Cells presented a rounded morphology, were multinucleated, displayed well-defined actin rings, and synthesized TRAP [64]. Exposure to the NPs had a similar effect on these parameters, and no significant differences were noted between the two particles. However, compared with the positive control and to the cultures treated with Mg(OH)_2_ NPs, Mg(OH)_2_RH particles modulated the expression of relevant osteoclastogenic genes. The pivotal transcription factor NFATC1, which regulates a number of osteoclastogenic genes (TRAP, cathepsin K, calcitonin receptor) [65], was downregulated. Furthermore, a similar effect was observed for the gene coding for CA2 and CTSK, involved in osteoclastic bone resorption [64]. Mg(OH)_2_RH particles have morphological features and crystallinity that favors their dissolution, compared with Mg(OH)_2_ NPs, thus probably yielding different levels of Mg ions. As it was observed with the osteoblasts, the Mg ion seems to have dose- and time-dependent effects on osteoclasts [66]. The presence of the bioactive molecules from the rosehip extract might be a contributing factor. The effect of rosehip bioactive compounds on osteoclasts has been poorly investigated, however, there is information mostly supporting a negative effect in osteoclastogenesis [32,54,56].

**Figure 6:**
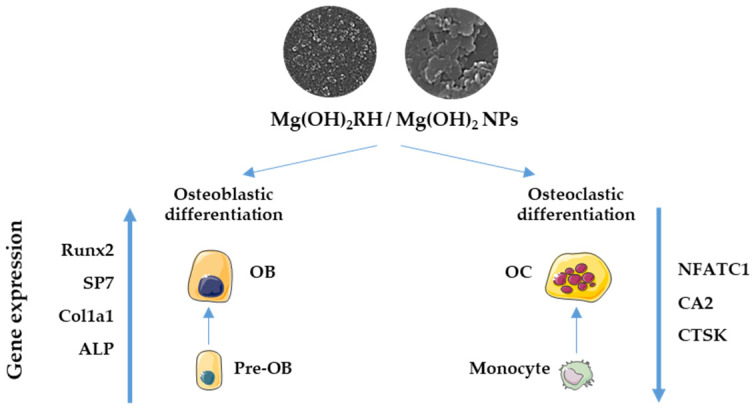
Schematic summary of the effect of Mg(OH)_2_ and Mg(OH)_2_RH NPs in gene expression of osteoblastic (OB) and osteoclastic (OC) cells. Mg(OH)_2_RH NPs increased osteoblastic differentiation by promoting the expression of Runx2, SP7, Col1a1, and ALP, and reduced osteoclastic differentiation by downregulating the expression of NFATC1, CA2, and CTSK.

The results concerning the effect of Mg(OH)_2_ and Mg(OH)_2_RH NPs on osteoblastic and osteoclastic cells are summarized in Figure 6. As a whole, the two NPs differ in morphology, size, and crystallinity, with these features favoring a higher cell/NPs interaction and dissolution rate for the RH-functionalized NPs. Furthermore, the presence of RH-bioactive compounds provided additional features to interact with bone cells. As mentioned above, mostly, experimental and clinical studies on rosehip’s biological properties converge to a positive effect on bone metabolism. RH contains high levels of flavonoids, a major subclass of polyphenols that have been shown to promote osteoblast differentiation, which ultimately results in bone formation [67]. Some of these compounds appear to inhibit osteoclastogenesis [68]. Still, there are also studies reporting different effects in these processes. Differences in the cell culture model, tested flavonoids, levels, and exposure time, evaluated parameters, and, importantly, the nature of the functionalized biomaterial, hinder the establishment of patterns. Nevertheless, reported information strongly supports the positive role of flavonoids in bone formation [68]. However, in the present work, with the used experimental protocol, the relative contribution of the particles physicochemical profile or the presence of the RH extract is not known.

In the present study, Mg(OH)_2_ NPs functionalized with the RH extract were tested in similar levels and exposure conditions in osteoblastic and osteoclastic cells—a methodological advantage that is not shared by most studies that only address one of the cell types. Mg(OH)_2_RH NPs exhibited higher osteoblastic differentiation potential, as evidenced by the increased ALP activity and expression of relevant genes. Furthermore, these NPs also reduced the expression of some osteoclastogenic genes. These observations suggest the potential usefulness of Mg(OH)_2_RH NPs in bone regeneration.

## Figures and Tables

**Table 1:** Genes and respective primers assay ID (BioRad) for RT-qPCR.

Gene	Gene Name	Assay ID
Reference	Glyceraldehyde-3-phosphate dehydrogenase (GADPH)	qHsaCED0038674
Osteoblastic	Runt-related transcription factor 2 (Runx2)	qHsaCED0044067
	SP7 transcription factor (SP7)	qHsaCED0003759
	Collagen type I alpha I chain (Col1α1)	qHsaCED0043248
	Alkaline phosphatase (ALP)	qHsaCED0045991
	Secreted protein acidic and rich in cysteine (SPARC), aka osteonectin	qHsaCID0010332
	Tumor necrosis factor receptor superfamily member 11b (TNFRSF11B), aka osteoprotegerin	qHsaCED0046251
Osteoclastic	Spi-1 proto-oncogene (SPI1)	qHsaCID0022097
	Nuclear factor of activated T cells 1 (NFATC1)	qHsaCED0044370
	Acid phosphatase 5, tartrate-resistant (ACP5)	qHsaCED0056724
	Carbonic anhydrase II (CA2)	qHsaCID0021039
	Cathepsin K (CTSK)	qHsaCID0016934

## Data Availability

The data presented in this study are available on request from the corresponding author.
